# Initial experience with pulsed field ablation for atrial fibrillation

**DOI:** 10.3389/fcvm.2022.959186

**Published:** 2022-11-08

**Authors:** Federico T. Magni, Bart A. Mulder, Hessel F. Groenveld, Ans C. P. Wiesfeld, Robert G. Tieleman, Moniek G. Cox, Isabelle C. Van Gelder, Tom Smilde, Eng S. Tan, Michiel Rienstra, Yuri Blaauw

**Affiliations:** ^1^Department of Cardiology, University of Groningen, University Medical Center Groningen, Groningen, Netherlands; ^2^Department of Cardio-thoracic Surgery, University of Groningen, University Medical Center Groningen, Groningen, Netherlands

**Keywords:** atrial fibrillation, posterior wall ablation, learning curve, pulsed field ablation, catheter ablation

## Abstract

**Introduction:**

Pulsed field ablation (PFA) was recently introduced for the treatment of symptomatic atrial fibrillation (AF) with the claim of selectively ablating the myocardium while sparing surrounding tissues. We present our initial experience with a PFA catheter for pulmonary vein isolation (PVI) and describe procedural findings and peri-procedural safety of the first 100 patients.

**Materials and methods:**

We investigated 100 patients treated for symptomatic AF using the FARAWAVE PFA catheter (Farapulse, Menlo Park, CA, USA) between July 2021 and March 2022. Procedure workflow and electrophysiological findings at the time of ablation, peri-procedural complications, and operator learning curves are described.

**Results:**

The mean age of patients was 62.9 ± 9.4 years, 62% were male subjects and 80% had paroxysmal AF. The median CHA_2_DS_2_-VASc score was 1.5 (IQR: 1.0–2.0) and the mean left atrial volume index was 35.7 ± 9.6 ml/m2. In 88 (88%) patients, PVI alone was performed and in 12 (12%) patients additional ablation of the posterior wall was performed. 3D-electroanatomic mapping was performed in 18 (18%) patients. Procedures without mapping lasted for 52.3 ± 16.6 min. The mean number of applications per pulmonary vein (PV) was 8.1 ± 0.6. In all patients (100%), all PVs were confirmed to be isolated. The learning curves of the two operators who performed > 20 procedures showed a negligible variation of performance over time and practice did not significantly predict procedure time [Operator 1 (senior): *R*^2^ = 0.034, *p* = 0.35; Operator 2 (junior): *R*^2^ = 0.004, *p* = 0.73]. There was no difference between the procedure times between senior and junior operators (Operator 1: 46.9 ± 9.7 min vs. Operator 2: 45.9 ± 9.9 min; *p* = 0.73). The only complications observed were two cases of bleeding at the site of percutaneous access.

**Conclusion:**

Our initial experience shows that use of the PFA catheter for pulmonary vein isolation (PVI) is safe, fast, and easy to learn.

## Introduction

Catheter ablation of the pulmonary veins (PVI) is the mainstay for the long-term treatment of symptomatic AF (1). PVI has been traditionally performed using thermal energy sources, such as radiofrequency ablation or cryoenergy ablation (1, 2). Both ablation technologies have shown excellent acute PVI rates, and long-term freedom of AF following ablation is comparable for both techniques in patients with paroxysmal AF (1, 2). However, severe complications (phrenic nerve palsy and atrio-oesophageal fistula) associated with the non-discriminatory nature of thermal injury still occur (1–4).

Recently, a new ablation technique has been introduced: pulsed field ablation (PFA). PFA is a non-thermal ablative modality leveraging ultrarapid electric fields which destabilize cell membranes of target tissues by forming irreversible nanoscale pores leading to leakage of cell contents and, eventually, apoptosis (5, 6). Importantly, the threshold for inducing cell death varies for different tissues (7, 8). This differential tissue sensitivity translates into the potential ability to perform full transmural lesions in the atrial myocardium while sparing adjacent tissues and structures. Although several pre-clinical studies investigated the safety and feasibility of PFA, limited reports have been published so far describing the clinical application of this novel catheter technology. In this study, we present our initial center experience with this novel ablation catheter and describe procedural findings and acute safety and efficacy in the first 100 patients treated with the PFA catheter for pulmonary vein isolation (PVI).

## Materials and methods

### Patient population

In this study, we investigated the first 100 patients treated for symptomatic AF using PFA at the University Medical Center Groningen (UMCG), Netherlands, between July 2021 and March 2022. Patients with paroxysmal, as well as persistent AF, were considered eligible for the procedure. All patients provided written informed consent. Pre-procedural investigations included trans-thoracic echocardiogram (TTE), cardiac computed tomography (CT) scan, electrocardiogram (ECG), and lab work. Pre-procedural TEE to exclude the presence of thrombi in the left atrial appendage (LAA) was performed in patients who had an inconclusive/no recent CT scan, CT scan with the suggestion of LAA thrombus, or when CHA_2_DS_2_-VASc score was higher than 2 in male subjects and 3 in female subjects. Anticoagulation was initiated at least 4 weeks before the procedure and continued for at least 3 months afterward.

### Procedure description

All procedures were performed using protocolized conscious sedation executed by a sedation specialist. Three femoral echo-guided venous punctures were performed to obtain venous access. A decapolar catheter was positioned in the coronary sinus. An ICE catheter (ViewFlex™ Xtra, Abbott, Chicago, IL, USA) was positioned in the right atrium for echo-guided transseptal puncture and for the visualization of ablation catheter contact with the pulmonary veins. Transseptal puncture was performed using an SL0 sheath. After a guidewire was positioned in the left superior PV, the SL0 sheath was exchanged for the Faradrive (Farapulse, Menlo Park, CA, USA) sheath and the Farawave (Farapulse) catheter was advanced into the left atrium. Heparin (100 IU/kg) was given prior to transseptal puncture, and the target activated clotting time (ACT) was > 300 s.

### Pulsed field ablation

**FIGURE 1:**
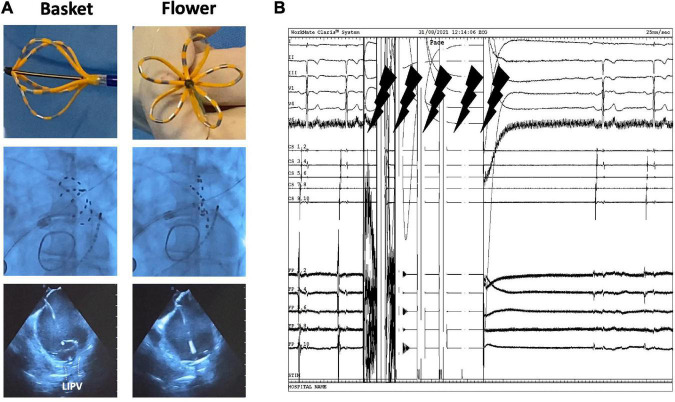
Pulsed field ablation system and workflow. **(A)** (In order from top to bottom) Physical, fluoroscopy, and echocardiogram view of the FARAWAVE catheter in both basket and flower shapes. **(B)** Electrograms showing biphasic waveform application with five pulses.

The PFA system had three components: a custom generator (Farastar, Farapulse) that delivered a high-voltage pulsed field waveform over multiple channels, a PFA catheter (31 or 35 mm diameter), and a 12-F steerable sheath (Faradrive, Farapulse). The 35 mm catheter size was used in patients with larger atria (LAVI > 40 ml/m2), left common ostium (LCO), or persistent AF. The 12-F over-the-wire PFA ablation catheter (Farawave, Farapulse) had five splines that each contain four electrodes and could be deployed in either a flower petal or basket configuration (Figure 1). When fully deployed into a flower pose, the maximum diameter of the distal portion was 31 or 35 mm, depending on the catheter size. The catheter was advanced over a guidewire until the splines achieved circumferential contact/proximity with the PV antra. To ensure contact between the catheter and PV ostium/antrum, we used fluoroscopy and/or ICE (Figure 1). On ICE, contact was assessed by observing visual contact of the catheter in the basket and flower positions (see Figure 1A), and on fluoroscopy when further advancement of the catheter was not possible (no contrast was used). The ICE was maneuvered in a clockwise fashion to bring into view the left and then the right pulmonary veins (9). The ablative energy was delivered from all electrodes; the third electrode on each spline could also record electrograms. The structure of the waveforms was a hierarchical set of microsecond-scale biphasic pulses, unsynchronized to cardiac rhythm (Figure 1). The catheter was rotated every two applications to ensure circumferential PV ostial and antral coverage. Four applications were given with the catheter in the basket shape and four additional applications were given in the flower shape. Each application consisted of five pulses. The application protocol for the LCO was dependent on the size of the LCO (assessed with ICE). For LCOs larger than the catheter’s diameter in the basket position, applications in the basket configuration were delivered in separate veins, and applications in the flower positions were delivered ideally at the LCO itself. If the LCO was narrower than the catheter’s diameter in the basket position, then applications in both basket and flower position were delivered at the LCO. Pre-and post-ablation electrograms were recorded with the Farawave (Farapulse) catheter positioned in the PV. Isolation after ablation was confirmed by the disappearance of PV potentials after ablation. Pacing at bipolar pairs (10 mA) was performed after ablation with the ablation catheter in the PV to verify the exit block. Of note, no contrast injections were used. For the isolation of the right-sided veins, no phrenic nerve pacing was performed. Following ablation, diaphragmatic movement was verified using fluoroscopy. When a vagal reaction occurred during ablation, leading to asystole (> 10 s), this was handled by administrating atropine.

### Posterior wall isolation

In a subgroup of patients, left atrial posterior wall isolation (PWI) was performed. For these procedures, 3D electroanatomic mapping was used (EnSite Precision™, Abbott, or Rhythmia HDx™, Boston Scientific, Marlborough, MA, USA) and the 3D anatomy was created with a high-density mapping catheter (Advisor™ HD grid, Abbott, or IntellaMap Orion™, Boston Scientific) or with the Farawave (Farapulse) catheter. After the isolation of every PV, the catheter in the flower shape—with the guidewire still in the PV ostium—was positioned against the posterior wall and two applications were delivered. This process was repeated for each PV. Subsequently, overlapping applications across the entire posterior wall were performed to ensure redundant coverage of the entire posterior wall. Before each application, the position of the Farawave^®^ catheter was depicted on the 3D map using a “shadow.” Following ablation, remapping was performed to verify the presence of a posterior box lesion. Pacing was performed for the exit block. No oesophageal temperature probe was used.

### Peri-procedural and secondary outcomes

Procedural findings include procedure time (from venous puncture to sheath removal), left atrial time (LA time: transeptal puncture to venous sheath removal), confirmed PV isolation, and number of applications per PV, as well as procedure-related complications during follow-up. Learning curves of operators who performed more than 20 procedures without mapping are described (Operator 1 and Operator 2). At the time of the study, Operator 1 had more than 10 years of experience (senior) and Operator 2 had less than 5 years of experience (junior). We provide a comparison of procedure times and radiation exposure with PVI-only cryoballoon ablations performed by the same operators involved in this study. The cryoballoon procedure description can be found in Supplementary material.

Procedure-related complications were classified into major and minor complications, in accordance with the consensus statement on surgical and catheter ablation and the classifications utilized by previous similar studies. Major complications included bleeding requiring thoracotomy or transfusion, permanent phrenic nerve paralysis, pacemaker device implantation, stroke/transient ischemic attack, atrio-esophageal fistula requiring surgery, and death. Minor complications included hemoptysis, pneumonia, and temporary phrenic nerve paralysis.

### Follow-up

Follow-up was scheduled for all patients to receive periodic ECG controls at the outpatient clinic at 3, 6, and 12 months after the procedure. A 72-h Holter monitoring was performed at 3, 6, and 12 months to check for the evidence of AF recurrence. If patients experienced AF-related symptoms, outpatient visits were scheduled before the upcoming follow-up visit.

### Statistical analyses

Patient characteristics, rate of complications, and procedure-related data are presented as mean and standard deviation for continuous variables and median and interquartile range or number and percentages for categorical variables. Spearman’s rank correlation was used to determine the correlation between practice (consecutive patients) and procedure time. The analyses were conducted using IBM SPSS Statistics for Windows, version 23 (IBM Corp., Armonk, NY, USA), and statistical significance was set at a *p*-value smaller than 0.05.

## Results

### Patient characteristics

Table 1 shows the baseline characteristics of the first 100 patients with symptomatic AF who were treated with PFA at our center. The mean age was 62.9 ± 9.4 years and most were men (62%). Prior to ablation, patients suffered predominantly from paroxysmal AF (80.0%). The median CHA_2_DS_2_-VASc score was 1.5 (1.0–2.0), and the mean left atrial volume index (LAVI) was 35.7 ± 9.6 ml/m^2^. Nine patients received PFA as a redo procedure following prior catheter ablation (3 radiofrequency and 6 cryoballoon) and in 8 of these 9, additional PWI was performed.

**TABLE 1:** Baseline characteristics.

Baseline characteristics	PFA (*n* = 100)*
Age	62.9 ± 9.4
Sex (M)	62% (62)
BMI	27.4 ± 3.6
Duration AF (months)	44.0 (16.6–95.6)
Type AF	
– Paroxysmal	80% (80)
– Persistent	18% (18)
– Long-standing persistent	2% (2)
Previous AF ablation	10% (10)
Heart failure	7% (7)
Cardiomyopathy	4% (4)
Coronary artery disease	19% (19)
Congenital heart disease	2% (2)
Pacemaker	2% (2)
Stroke/TIA	4% (4)
Thromboembolic event	7% (7)
COPD	3% (3)
Vascular disease	3% (3)
Renal failure	3% (3)
OSAS	3% (3)
Hypertension	37% (37)
Diabetes mellitus	7% (7)
Family history of AF	3% (3)
Failed AAD1	36% (36)
Failed AAD2	39% (39)
Failed AAD3	29% (29)
Failed AAD4	29% (29)
LVEF [%]	54.7 ± 3.7
LAVI [mL/m^2^]	35.7 ± 9.6
CHA_2_DS_2_-VASc score	1.5 (1.0–2.0)

BMI, body mass index; AF, atrial fibrillation; TIA, transient ischemic accident; COPD, chronic obstructive pulmonary disease; OSAS, obstructive sleep apnea syndrome; AAD, antiarrhythmic drug; LVEF, left ventricular ejection fraction; LAVI, left atrial volume index.

*Data are presented as mean and standard deviation for continuous variables and median and interquartile range or number and percentages for categorical variables.

### Procedural findings

Procedural findings are shown in Table 2. The 31 and 35 mm catheters were used in 77% (77) and in 23% (23) of procedures, respectively. In 88 patients, PVI alone was performed. The remaining 12 patients underwent additional PWI. Overall, the mean procedure time was 60.9 ± 26.8 min, and the mean radiation time and dose were 13.5 ± 7.5 min and 658.0 (376.0–1037.0) μGy/m^2^, respectively. Procedures without 3D mapping lasted a mean of 52.3 ± 16.6 min. 3D Mapping was performed in six patients during the early experience for PVI-only ablation, and later mapping was exclusively in patients scheduled for PVI + PWI (10), eight of which were redo cases after prior cryoballoon/RF ablation. In total, 3D electroanatomic mapping was performed during 18 procedures, with a mean procedure time of 105.5 ± 25.5 min and mean radiation time and dose of 16.0 ± 5.1 min and 731.6 (563.0–1265.0) μGy/m^2^, respectively.

**TABLE 2:** Procedural findings.

Procedural findings	PFA (*N* = 100)*
N^o^ procedures performed	
– Operator 1	37% (37)
– Operator 2	35% (35)
– Operator 3	14% (14)
– Operator 4	6% (6)
– Operator 5	4% (4)
– Operator 6	4% (4)
Mapping	18% (18)
Posterior wall isolation	12% (12)
– Average applications	19.2 ± 8.7
Procedure time (skin to skin) [min]	
– With mapping	105.5 ± 25.5
– Without mapping	52.3 ± 16.6
– Total	60.9 ± 26.8
LA time [min]	44.8 ± 23.5
Same day discharge	81% (81)
Radiation time [min]	13.5 ± 7.5
Radiation dose [μGy/m^2^]	658 (376–1037)
RF touch up	0% (0)
Isolated PVs (isolated/total PVs)	100% (391/391)
Total applications	32.3 ± 2.5
Average applications per PV	8.1 ± 0.6
– RSPV	8.2 ± 1.0
– RIPV	8.1 ± 0.5
– LSPV	8.3 ± 1.2
– LIPV	8.2 ± 0.8
– LCO	11.1 ± 2.7
PFA catheter	
– 31 mm	77% (77)
– 35 mm	23% (23)

LA, left atrium; RF, radiofrequency; PV, pulmonary vein; RSPV, right superior PV; RIPV, right inferior PV; LSPV, left superior PV; LIPV, left inferior PV; LCO, left common ostium; PFA, pulsed field ablation.

*Data are presented as mean and standard deviation for continuous variables and median and interquartile range or number and percentages for categorical variables.

For the 72 cryoballoon procedures performed by five (Operators 1, 2, 3, 5, and 6) of the six operators who performed the PFA procedures in this study, the mean procedure time for PVI was 74.5 ± 21.7 min and the mean LA time was 59.0 ± 19.7 min. Mean radiation time and median radiation dose were 14.1 ± 9.6 min and 985.5 (306.2–1652.1) μGy/m^2^, respectively.

The independent sample *t*-test showed significantly lower mean procedure time—of PVI-only procedures without mapping—(PFA: 52.6 ± 16.6 min vs. cryoballoon: 74.5 ± 21.7 min; *p* < 0.001) and lower mean LA time (PFA: 37.1 ± 14.1 vs. cryoballoon: 59.0 ± 19.7; *p* < 0.001) with PFA compared to cryoballoon ablation. The Mann–Whitney *U* test showed no significant difference in radiation dose between PFA and cryoballoon [PFA: 985.5 (306.2–1652.1) μGy/m^2^ vs. cryoballoon: 985.5 (306.2–1652.1) μGy/m^2^; *p* = 0.152]. No significant difference was shown in fluoroscopy time (PFA: 13.0 ± 7.9 vs. cryoballoon: 14.1 ± 9.6; *p* = 0.425).

**FIGURE 2:**
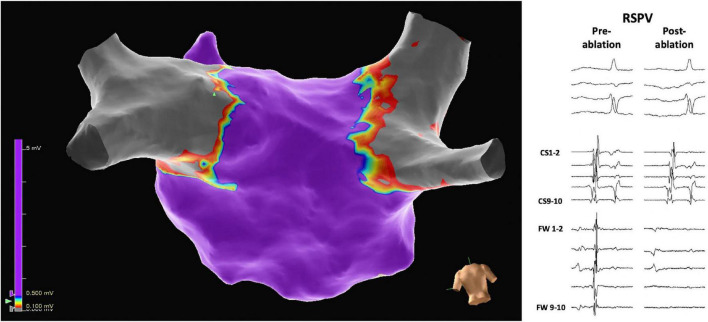
Three-dimensional electrophysiological voltage maps after pulmonary vein isolation were performed with pulsed field ablation. On the right, pre-and post-ablation electrograms demonstrate the isolation of, in this case, the right superior pulmonary vein (RSPV). The scale of the electrograms shown is 100 mm/sec.

**FIGURE 3:**
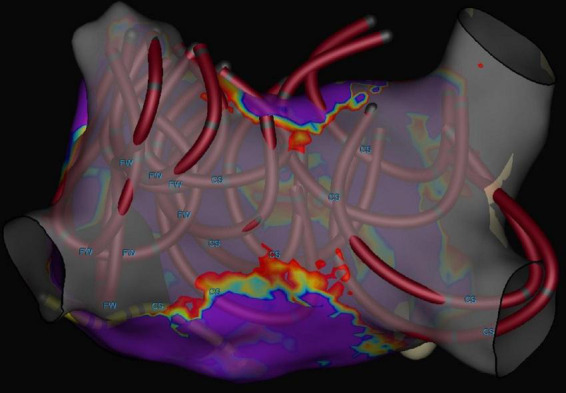
Three-dimensional electrophysiological voltage maps after pulmonary vein isolation + posterior wall ablation performed with pulsed field ablation. The circular catheters show the locations where PFA was applied with a catheter in the flower position.

The mean number of applications per PV was 8.1 ± 0.6. Nine patients had a left common ostium, for which the mean number of applications was 11.1 ± 2.7. In all patients, all PVs (100%) were confirmed to be isolated at the end of ablation (Figure 2). No radiofrequency catheter touch up was performed. Figure 3 shows a 3D electroanatomic map of the left atrium after PVI and PWI and the shadows show the location of the ablation catheter during each application. The average number of PFA applications to the posterior wall was 19.2 ± 8.7.

### Learning curve

**FIGURE 4:**
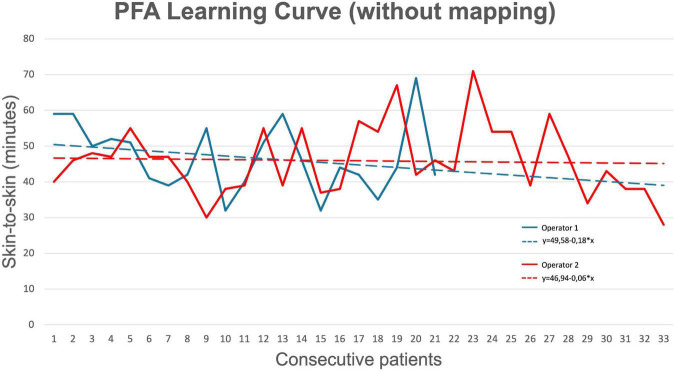
Learning curve for pulsed field ablation (of operators who performed > 20 procedures). Operator 1 (senior) had more than 10 years of experience with AF ablation, while Operator 2 (junior) had less than 5 years of experience with AF ablation.

Six different operators performed the ablations described in this study: Operator 1 performed 37 procedures (37%); Operator 2 performed 35 procedures (35%); Operator 3 performed 14 procedures (14%); Operator 4 performed six procedures (6%); Operator 5 performed four procedures (4%); and Operator 6 performed four procedures (4%). Figure 4 shows the learning curves for Operators 1 and 2 who both performed more than 20 procedures without mapping. The comparison showed no significant difference between the mean procedure times of the two operators (Operator 1: 46.9 ± 9.7 min; Operator 2: 45.9 ± 9.9 min; *p* = 0.73). In addition, Spearman’s rank correlation showed that practice was not correlated with procedure time (Operator 1: correlation coefficient = −0.246, *p* = 0.283; Operator 2: correlation coefficient = −0.139, *p* = 0.439).

### Early follow-up findings

Three-month follow-up was available for 76 patients (76%). The median follow-up was 90.5 (83–98) days. All but one patient had rhythm monitoring (24-h Holter at 3 months visit or implantable loop recorder) which revealed early recurrence of AF in six (6%) patients and atypical flutter in two (2%) patients. Two patients underwent electrical cardioversion due to a recurrence of AF during the blanking period.

### Safety

Table 3 summarizes the complications observed in our cohort during the available follow-up period. No patients suffered major complications during or after the ablation. Two patients (2%) suffered bleedings at the percutaneous access sites of the femoral vein after the procedure. This was managed by applying pressure and compression bandages.

**TABLE 3:** Rate of procedural complications.

Adverse events	PFA (*N* = 100)
Major complications^†^	
– Death	0% (0)
– TIA/CVA	0% (0)
– Atrio-oesophageal fistula	0% (0)
– Permanent phrenic nerve palsy	0% (0)
– Bleeding requiring intervention*	0% (0)
Minor complications^†^	
– Temporary phrenic nerve palsy	0% (0)
– Access site bleeding	2% (2)
**Total**	2% (2)

*Thoracotomy or transfusion.

^†^In accordance with the consensus statement on surgical and catheter ablation (2) and the classifications utilized by previous similar studies.

## Discussion

In this study, we presented our initial experience with a PFA catheter for PVI for the treatment of symptomatic AF in 100 patients. We observed that PFA is a safe ablation modality with effective acute isolation of the PVs. Procedure times were short since the early phases of the learning curve, and comparable between operators with different levels of experience. In addition, ablation beyond the PVs (left atrial posterior wall ablation) is feasible.

### Pulsed field ablation so far: Procedural findings and efficacy

Over the last decade, pre-clinical studies investigating PFA for the ablation of AF in animal models showed favorable safety profiles and lesion durability (11). Histological assessment of the treated myocardium confirmed that PFA achieved transmural lesions with superior durability (biphasic = 100%; monophasic = 55.6%; RFA = 50%; *P* = 0.002) and greater organization of fibrotic tissue compared to RFA lesions, all while sparing adjacent and surrounding structures (11).

To date, few studies have reported the use of PFA in the clinical setting for the treatment of symptomatic AF. These utilized different catheters and waveform protocols which makes the comparison of outcomes difficult.

The first clinical application was described by Reddy et al., who used PFA to perform endocardial PVI on 15 patients with paroxysmal AF (12). Acute isolation was achieved in 100% of the PVs after 12.4 ± 1.0 applications per patient (3.26 ± 0.5 applications/PV), with a total average procedure time of 67 ± 10.5 min. In 2020, the same group reported the 1-year outcomes of the first-in-human, non-randomized feasibility trials, which enrolled a total of 121 patients across three centers (10). Acute PVI was achieved in 100% of patients after a mean of 7.2 applications per PV, with a mean procedure time of 96.2 ± 30.3 min (including mapping). Re-mapping after ablation revealed durable PVI in 84.1% of patients, following the optimization of the pulsed field waveform. At 1 year, 81.1% of patients were free from AF. A similar acute PVI rate was shown in a recent study using a variable loop catheter to perform PFA, which also reported 100% acute PVI in all 10 patients included (13). A recent study by Kueffer et al. investigated a multipolar PFA catheter in 56 patients (14). Acute PVI assessed using the PFA catheter was achieved in 100% of PVs, while secondary assessment using high-density 3D mapping revealed isolation in 93% of PVs. Following additional applications, 100% of PVs were confirmed to be isolated. In line with all these studies, we also achieved 100% acute PVI with a similar number of applications per PV [for those who used the Farawave (Farapulse) catheter] and showed freedom from AF/AFL during early follow-up in 82% of patients.

Only one study investigated PFA for the treatment of persistent AF, in 25 patients who received PVI, PWI, and cavo-tricuspid isthmus line (15). Re-mapping at 3 months after ablation confirmed durable PVI, PWI, and cavo-tricuspid isthmus line in 96%, 100%, and 100% of the cases, respectively. An early report of the long-term outcomes of this series showed a 92 ± 5.4% 1-year freedom from atrial tachyarrhythmias (16). In line with this study, we also showed that ablation beyond the PVs is feasible with PFA (posterior wall ablation).

### Safety profile and tissue selectivity

The distinguishing feature of PFA is the alleged ability to selectively ablate the myocardium without damaging adjacent tissues and structures (e.g., phrenic nerve palsy and atrio-oesophageal fistula) (6–8, 10–13, 15). In agreement with findings from previous studies, we observed only two minor complications (both bleedings from the femoral access site). Of note, we did not observe any phrenic nerve palsy, nor did we observe clinical evidence of oesophageal involvement. One patient suffered a vagal reaction during the ablation of the LSPV, leading to asystole which was handled by administrating atropine. One study reported ST elevation following PFA application, (13) and another study reported coronary spasm following PFA application at the mitral isthmus (17). Furthermore, other safety concerns encountered with conventional ablation modalities, such as silent gas emboli, seem to persist with PFA but the evidence is lacking thus far (13, 18, 19). Therefore, additional targeted safety studies are warranted.

### Learning curve: Pulsed field ablation vs. thermal techniques

In addition to favorable safety and efficacy profiles, PFA appears to be associated with quick procedure times and short learning curves. In our study, we observed a mean procedure time without the mapping of 52.3 ± 16.6 min and with mapping of 105.5 ± 25.5 min. Most previous studies performed mapping as standard and procedure times are comparable with ours where mapping was performed. Our findings also highlight that PFA is associated with a very short learning curve. Since the earliest interventions, operators achieved quick procedure times, without compromising the rate of acute PVI. Furthermore, results were consistent and comparable between operators with different levels of experience (Operator 1: > 10 years, Operator 2: < 5 years). Indirect comparison with radiofrequency and cryoballoon ablation highlights slower procedure times (mean procedure time: 124–141 min) and longer learning curves with the thermal ablation modalities compared to PFA (20). Direct comparison with cryoballoon PVI procedures performed at our center revealed significantly lower procedure time and LA time with PFA compared to cryoballoon ablation (*p* < 0.001). The fast uptake of the correct PFA handling and over-the-wire technique may have been facilitated by the experience operators already possessed with cryoballoon ablation.

### Limitations

In the present observational study, we provide an early report on our first experience with PFA for the treatment of symptomatic AF but had only a small sample and limited follow-up. Therefore, we cannot draw conclusions on the mid or long-term efficacy of PFA. In addition, the lack of remapping data after ablation prevents us from drawing conclusions on the durability of PFA lesions (10). The operators included in this study were already experienced with one-shot cryoballoon ablation. This may have impacted our learning curves, with rather short procedure times since the earliest cases and no significant improvement. Additionally, we monitored for the occurrence of major and minor complications (see Table 3) but no targeted investigations were carried out for the detection of complications, such as silent gas emboli, PV stenosis, or oesophageal involvement (18).

### Future studies and implications

Future studies should investigate the long-term freedom from AF and lesion durability of PFA, in order to draw more stringent conclusions on PFA’s long-term efficacy. Randomized controlled trials comparing PFA to conventional thermal ablative technologies will be necessary to show differences between these two ablation modalities. Larger studies providing targeted safety investigations are necessary to confirm the favorable safety findings observed in our and previous studies and provide greater clarity on the safety of PFA.

## Conclusion

In this single-center study, we report our initial experience with the PFA catheter for PVI in 100 patients. In this initial series, we observed short learning curves and no signs of serious complications. Future studies are warranted to determine long-term efficacy and safety outcomes.

## Data availability statement

The raw data supporting the conclusions of this article will be made available by the authors, without undue reservation.

## Ethics statement

Dutch law allows the use of electronic health care records for research purposes under certain conditions. According to this legislation, neither obtaining informed consent from patients nor approval by a Medical Ethics Committee is obligatory for this type of observational studies containing no directly identifiable data (Dutch Civil Law, Article 7:458).

## Author contributions

All authors listed have made a substantial, direct, and intellectual contribution to the work, and approved it for publication.
