# Supplementary material for: Initial experience with pulsed field ablation for atrial fibrillation
Source: Front Cardiovasc Med. 2022 Nov 8;9:959186. doi: 10.3389/fcvm.2022.959186 (PMC9679623; doi:10.3389/fcvm.2022.959186)
Supplement: Supplementary file 1 [file Data_Sheet_1.docx]

**Appendix 1. Cryoballoon ablation procedure description**

The left atrium was accessed with a single trans-septal puncture after which 100E/kg bolus dose of unfractionated heparin was administered (target activating clotting time >300s). Pulmonary vein (PV) angiography was used to identify the PVs. Routinely a 28mm cryoballoon was used and delivered to the atrium via a 12Fr steerable sheath (FlexCath®; Medtronic, Inc.). Cryoapplication was delivered at the antrum of the PVs when PV occlusion was demonstrated with the aid of fluoroscopy and radiopaque contrast agent. Initially, at least two cryoapplications of 240 sec were delivered per vein. From end 2017 cryoapplication dosing was delivered according to the methodology described by Aryana et al.(1) The acute procedural end point was defined as the disappearance and absence or dissociation of all PV potentials using a 20mm circular mapping catheter (Achieve, Medtronic). Phrenic nerve pacing was performed during ablation of the right sided PVs.

1. Aryana A, Mugnai G, Singh SM, Pujara DK, de Asmundis C, Singh SK, et al. "Procedural and biophysical indicators of durable pulmonary vein isolation during cryoballoon ablation of atrial fibrillation." Heart Rhythm 13.2 (2016): 424-432. 10.1016/j.hrthm.2015.10.033.
